# CD44 and CLDN3 as immune-metabolic regulators in acute pancreatitis: a multi-modal transcriptomics study and experimental validation

**DOI:** 10.3389/fimmu.2025.1665200

**Published:** 2025-10-22

**Authors:** Xinwei Wang, Cheng Hu, Tian Liu, Rui Yang, Yuxin Shen, Shihang Zhang, Lihui Deng, Qing Xia

**Affiliations:** West China Centre of Excellence for Pancreatitis, Institute of Integrated Traditional Chinese and Western Medicine, West China Hospital, Sichuan University, Chengdu, China

**Keywords:** acute pancreatitis, inflammation, glycolysis, metabolic disorder, immune cell infiltration, machine learning, cellular landscape

## Abstract

Acute pancreatitis (AP) is an inflammatory disorder of exocrine pancreas regulated by a complex interaction between injured pancreatic acinar cells and immune cells. Recent studies indicated the crucial role of glycolysis in regulating immune cell function and inflammation. Here, we identified 43 glycolysis-related differentially expressed genes (DEGs) from transcriptomic datasets (GSE65146 and GSE109227). Through three machine learning algorithms,Claudin-3 (CLDN3) and CD44 were identified as key glycolysis-related DEGs. Their significant upregulation was further validated in an independent dataset. Then, single-sample gene set enrichment analysis revealed CLDN3 and CD44 were significantly correlated with immune-related structural remodeling and immune infiltration patterns. Single-cell RNA-seq analysis from GSE279876 confirmed that CLDN3 was downregulated in acinar cells, while CD44 was enriched in ductal and immune cells. To validate these findings, we established an AP model by 10 hourly intraperitoneal injections of caerulein (100 μg/kg) combined with one injection of lipopolysaccharide (10mg/kg). We confirmed that CD44 was upregulated and primarily expressed in inflammatory cells in AP mice. Interestingly, while CLDN3 mRNA levels were increased, its protein expression was reduced. Immunohistochemistry further revealed a redistribution of CLDN3 from the apical membrane to the cytoplasm in the pancreas of AP mice. Our findings, for the first time, indicated that CD44 and CLDN3 were crucial biomarkers associated with immune-metabolic dysregulation between pancreatic acinar cells and immune cells. The results of this study showed the potential of these two biomarkers as therapeutic targets for AP.

## 1 Introduction

Acute pancreatitis (AP) is one of the most common digestive disorders worldwide and imposes a growing global health burden (1, 2). Up to 20% of AP patients progress to severe acute pancreatitis, which is associated with a mortality rate of 25% to 35% (3). As the molecular mechanisms of AP remain poorly understood, there is a lack of reliable early biomarkers and targeted therapies.

The crosstalk between pancreatic acinar cells and immune cells drives the disease progression. Cumulative evidences demonstrate that AP is primarily initiated by the premature activation of digestive enzymes within acinar cells (4). The subsequent release of proinflammatory cytokines and chemokines from the damaged acinar cells recruits the immune cells to the pancreas (5–8). The local inflammatory response of the pancreas further drives aberrant activation of adaptive immune responses (9, 10) to exacerbate the inflammation, triggering systemic inflammatory response and multiple organ dysfunction. Recent advances (8, 11–17) in single-cell RNA sequencing (scRNA-seq) have provided valuable insights into the cellular heterogeneity and immunopathology of AP. These studies have identified distinct neutrophil (12, 18, 19) and macrophage subpopulations (20–22) and uncovered immune–stromal interactions (18, 19, 23, 24) that drive both local pancreatic injury and systemic complications, highlighting the pivotal role of immune remodeling in AP progression.

Glycolysis is a cytosolic metabolic pathway that generates a rapid source of energy in the form of adenosine 5’-triphosphate (ATP) and nicotinamide adenine dinucleotide from the conversion of glucose by a cascade of enzymatic reactions. Beyond its traditional role in the anaerobic production of ATP, recent studies (25–29) have revealed that glycolysis also functions as a multifaceted metabolic pathway and signaling hub, which plays a crucial role in regulating the functions of immune cells and inflammatory response. Activated immune cells, including macrophages, B cells, and T cells, undergo a metabolic shift towards glycolysis to fuel processes of cytokine secretion, proliferation, and migration (30). However, the roles of glycolysis in both signaling and metabolic processes in AP have previously been overlooked.

A better understanding of the underlying molecular mechanisms of glycolysis in AP is essential to identify the promising biomarkers and therapeutic strategies. This study aims to systematically identify crucial glycolysis-related differentially expressed genes (DEGs) in AP through integrative transcriptomic analysis, to explore their functional roles, regulatory mechanisms, and immunometabolic relevance, and to validate the findings in the experiments *in vivo*.

## 2 Materials and methods

### 2.1 Data acquisition, differential expression analysis, and functional enrichment analysis

**Figure 1:**
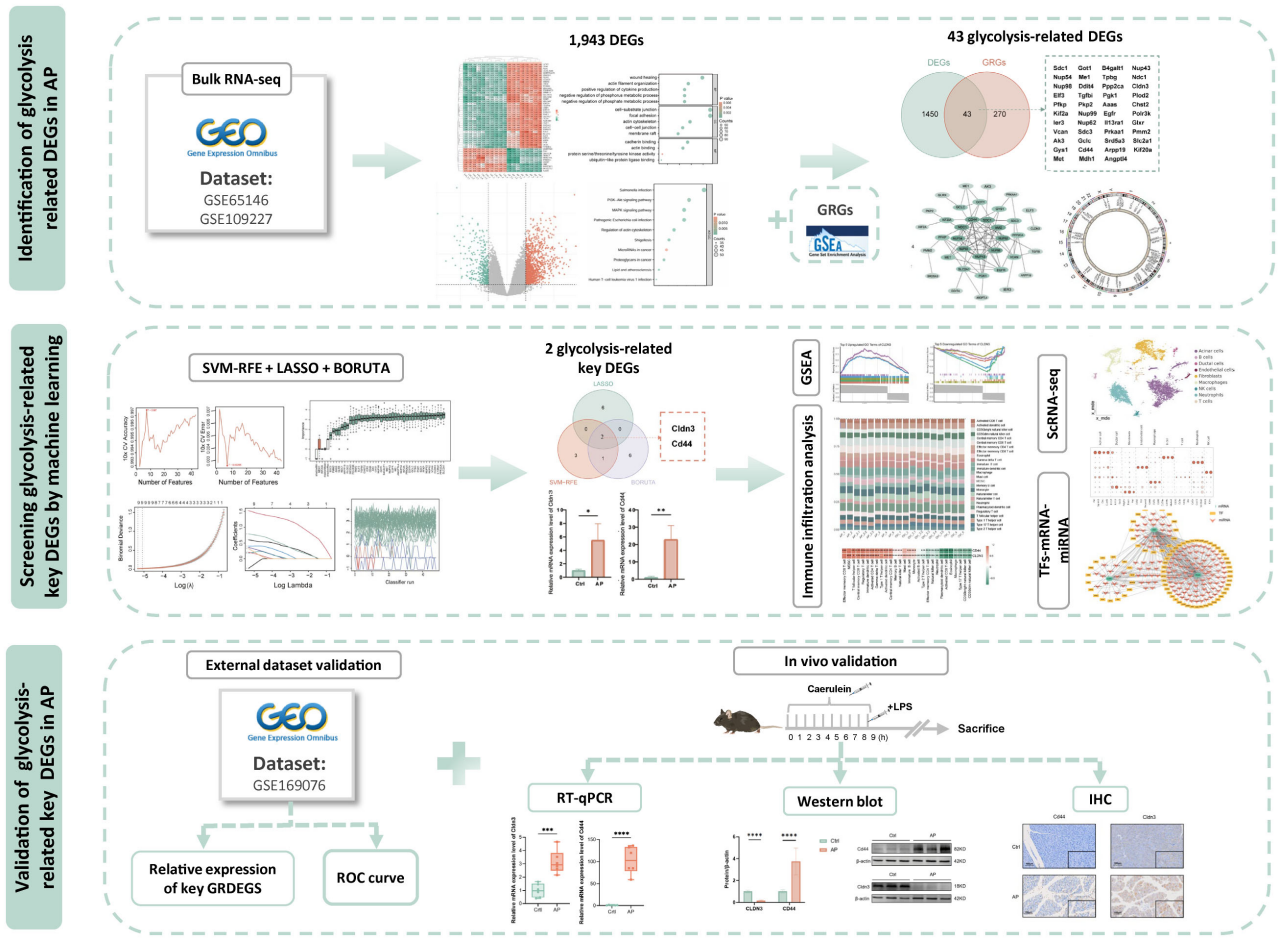
Study flowchart. DEGs, differentially expressed genes; AP, acute pancreatitis;GRGs, glycolysis related genes.

All datasets were obtained from the Gene Expression Omnibus (https://www.ncbi.nlm.nih.gov/geo/). The microarray datasets GSE65146 and GSE109227, based on the GPL6246 platform, were used for transcriptome analysis of pancreatic tissues from AP and control mice. Batch effects were corrected, and DEGs between AP and CTRL were identified using the limma package in R (version 4.3.1). Genes were defined as differentially expressed if |(logFC)| > 1 and adjusted P-value < 0.05. Principal component analysis (PCA) was conducted to assess sample clustering and to evaluate the effectiveness of batch effect correction. The datasets GSE169076 (GPL23479) and GSE298193 (GPL25947) served as independent external validation cohorts in this study. The single-cell RNA-seq dataset GSE279876 (GPL19057) was used for cell-level resolution analysis. Detailed dataset characteristics and group assignments are provided in **Supplementary Table 1**. To explore the biological functions and pathways associated with the DEGs, Gene Ontology (GO) enrichment and Kyoto Encyclopedia of Genes and Genomes (KEGG) pathway analyses were performed using the clusterProfiler package in R. Terms or pathways with a P-value < 0.05 were considered significantly enriched.The overall workflow of this study is illustrated in **Figure 1**.

### 2.2 Identification of the key glycolysis-related DEGs by machine-learning algorithms

Glycolysis-related genes were compiled from gene sets in the Molecular Signatures Database (https://www.gsea-msigdb.org/gsea/msigdb/). After harmonizing gene symbols and removing duplicates (**Supplementary Table 2**), Glycolysis-related genes were intersected with the DEGs identified in our dataset to create a subset for downstream analysis. Three complementary machine learning algorithms including LASSO regression, Boruta feature selection, and SVM-RFE. LASSO logistic regression was performed using the glmnet R package and 10-fold cross-validation to select features based on the optimal lambda values (lambda.min and lambda.1se). Boruta in the Boruta R package was utilized to assess feature importance over 50 iterations, and tentative features were refined using TentativeRoughFix(). SVM-RFE was conducted using the sigFeature package and a custom msvmRFE script with 10-fold cross-validation to select the most predictive genes iteratively. Final key genes were defined by the intersection of the results from all three methods and validated by ROC curve analysis in the validation dataset.

### 2.3 Gene set enrichment analysis

Gene Set Enrichment Analysis (GSEA) was performed using the “HALLMARK_GLYCOLYSIS” gene set obtained from the MSigDB database to explore the functional relevance of glycolysis-related phenotypes. The analysis was based on a ranked list of all expressed genes ordered by their differential expression statistics. GSEA was conducted using the clusterProfiler R package. Enrichment significance was evaluated by normalized enrichment scores (NES) and false discovery rate (FDR) values. To further investigate the functional roles of key glycolysis-related DEGs, we performed GSEA on GO biological process terms for each gene individually. Genes with an absolute Spearman correlation ≥ 0.8 and p-value < 0.05 were selected for downstream analysis. Based on NES, the top five significantly enriched upregulated and downregulated GO biological processes were identified.

### 2.4 Protein-protein interaction network construction

Protein-protein interaction (PPI) networks were constructed to explore potential functional relationships among the glycolysis-related DEGs. Interaction data were obtained from the STRING database (https://cn.string-db.org/). Network visualization and analysis were carried out using Cytoscape (version 3.9.1). Hub genes were identified using the Maximal Clique Centrality (MCC) algorithm implemented in the CytoHubba plugin.

### 2.5 Single-cell RNA-seq analysis

ScRNA-seq data from control and AP samples (GSE298193) were processed using Scanpy (Python 3.8). Cells with < 200 genes, < 1,000 UMIs, or > 5% mitochondrial reads were excluded to remove low-quality or dying cells, and genes detected in fewer than 3 cells were removed to reduce noise. Putative doublets were identified using Scrublet (31), which simulates artificial doublets from the observed data and classifies cells based on transcriptome similarity. Scrublet was run separately for each sample with automatic thresholding, and cells predicted as doublets were removed before downstream analyses. Counts were normalized with a delta-method shifted log transformation, and the 2,000 most highly variable genes (HVG) were selected via the Pearson-residual method. PCA was performed on the scaled HVG matrix (top 50 PCs retained), and Leiden clustering was applied to the PCA space (resolution = 0.25). Cluster identities were assigned manually based on marker genes curated from CellMarker 2.0 (http://117.50.127.228/CellMarker/) and PanglaoDB (https://panglaodb.se/index.html). For visualization, a modified distance embedding was computed from the PCA space.

### 2.6 Immune cell infiltration analysis

The levels of immune cell infiltration were estimated using single-sample Gene Set Enrichment Analysis (ssGSEA) based on immune cell marker gene sets (32)(**Supplementary Table 4**). The enrichment scores were normalized to calculate the relative proportions of immune cells across samples. Differences in immune infiltration between groups were evaluated using Student’s t-test with multiple testing correction. Spearman correlation analysis was conducted to assess relationships among immune cell types as well as between immune infiltration and the key glycolysis-related DEGs. The results were visualized using heatmaps and boxplots to depict patterns of immune cell infiltration and their associations with core gene expression.

### 2.7 TFs-mRNA- miRNA regulatory network construction

NetworkAnalyst (https://www.networkanalyst.ca/) was used to generate the regulatory networks. TF-gene interactions were derived from ENCODE ChIP-seq data applying the BETA Minus algorithm with a peak intensity signal threshold of <500 and a predicted regulatory potential score of <1. MiRNA–gene interactions were obtained from miRTarBase v9.0. The integrated TF–miRNA–mRNA regulatory relationships were visualized using Cytoscape.

### 2.8 Experimental model, histological assessment

C57BL/6 mice (6–8 weeks old, 18–24 g weight) were purchased from Jiangsu GemPharmatech Co., Ltd. and housed under specific pathogen-free conditions (22–24 °C, 50–60% humidity, 12 h light/dark cycle). The AP model was induced by 10 hourly intraperitoneal (i.p.) injections of caerulein (100 μg/kg; Tocris, UK, Cat No.6264). Lipopolysaccharide (LPS; 10mg/kg; Sigma, USA, Cat No.2880) was administered (i.p.) immediately after the 10th caerulein injection. Mice were sacrificed 12 h after LPS injection. Amylase and lipase in serum and myeloperoxidase (MPO) activity in pancreatic and lung tissues were measured. Specimens of pancreatic and lung tissue were fixed in 10% formalin at room temperature for 24 hours and embedded in paraffin. Sections were stained with hematoxylin and eosin (H&E). Pathological severity was semi-quantitatively scored based on edema, inflammatory infiltration, and acinar necrosis, each rated on a 0–3 scale, as previously described (33, 34). Lung injury assessment was conducted by quantifying edema and the accumulation of inflammatory cells in 10 randomly selected fields per section at 100× magnification, as described in previous studies (33). Whole-slide imaging was performed using the Olympus SLIDEVIEW VS200 system (Olympus, Japan), and Fiji software was used for digital image analysis.

### 2.9 RT-qPCR analysis

Total RNA was extracted from pancreatic tissues using TRIzol reagent and was reverse transcribed into cDNA. qPCR was conducted using the Bio-Rad CFX96 Real-Time PCR System (Bio-Rad, USA) and SYBR Green Master Mix (Vazyme, China). Gene expression was quantified by the 2^^−ΔΔCt^ method, normalized to 18S rRNA. Reagents and primer sequences are provided in the **Supplementary Materials**.

### 2.10 Western blot and immunohistochemistry

Western blot and immunohistochemistry (IHC) were performed to detect CLDN3 and CD44 expression in pancreatic tissues following standard protocols. The detailed methods were described in the **Supplementary Materials**.

### 2.11 Statistical analysis

Statistical analyses were conducted using R software (version 4.3.1) and GraphPad Prism 9.5 (GraphPad Software Inc., USA). Normally distributed data were presented as mean ± standard deviation and were analyzed using Student’s t-test or one-way analysis of variance. Continuous data were analyzed using the Mann–Whitney U test. Correlations between variables were assessed using Pearson’s or Spearman’s correlation coefficients. All tests were two-tailed, and p < 0.05 was considered statistically significant.

## 3 Results

### 3.1 Identification of differential expression of genes of AP

**Figure 2:**
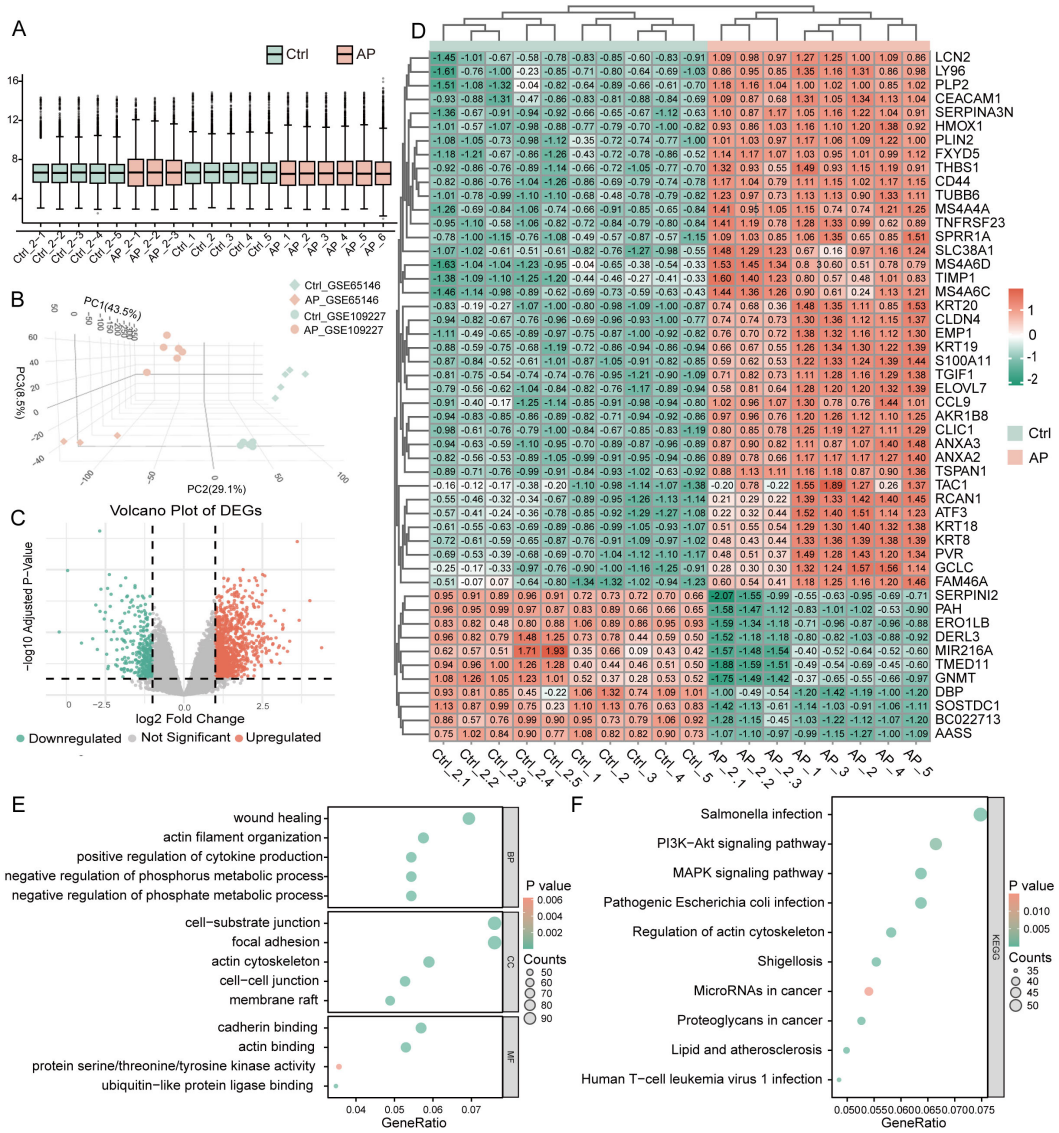
Identification and functional annotation of DEGs in AP. **(A)** Boxplot illustrating the distribution of gene expression values across all samples after batch effect correction. **(B)** The three-dimensional Principal Component Analysis plot shows AP and control sample clustering. **(C)** Volcano plot of DEGs between AP and control samples (threshold: |log_2_FC| ≥ 1, P < 0.05). **(D)** Heatmap of the top 50 DEGs ranked by statistical significance. **(E)** GO enrichment analysis of DEGs visualized using bubble plots, covering BP, CC, and MF categories. **(F)** KEGG pathway enrichment analysis of DEGs, shown as bubble plots. DEGs, differentially expressed genes; AP, acute pancreatitis; Ctrl, control; GO, Gene Ontology; BP, biological process; CC, cellular component; MF, molecular function; KEGG, Kyoto Encyclopedia of Genes and Genomes.

After batch correction, normalized expression values were evenly distributed across the 10 Ctrl (Control) and 9 AP samples (**Figure 2A**; **Supplementary Figure 1**). Three-dimensional PCA confirmed a clear separation between AP and Ctrl samples (**Figure 2B**). Differential expression analysis using the limma package (|log_2_FC| ≥ 1, adjusted P < 0.05) identified 1,943 DEGs, comprising 1,156 upregulated and 787 downregulated genes (**Figure 2C**). Hierarchical clustering of the top 50 DEGs clearly distinguished AP from Ctrl samples, further supporting the differential expression patterns (**Figure 2D**). GO enrichment revealed that these DEGs are predominantly involved in biological processes (BP) such as wound healing, actin-filament organization, and positive regulation of cytokine production; associated with cellular components (CC) including the cell–substrate junction, focal adhesion, and actin cytoskeleton; and exhibited molecular functions (MF) such as cadherin binding, actin binding, and protein serine/threonine/tyrosine kinase activity (**Figure 2E**). KEGG pathway analysis showed significant enrichment in the PI3K–AKT, and MAPK signaling pathways (**Figure 2F**). These pathways reflect key pathophysiological features of AP, including immune activation, inflammatory signaling, cellular stress responses, and tissue repair mechanisms.

### 3.2 Glycolysis pathway enrichment and identification of glycolysis-related DEGs in AP

**Figure 3:**
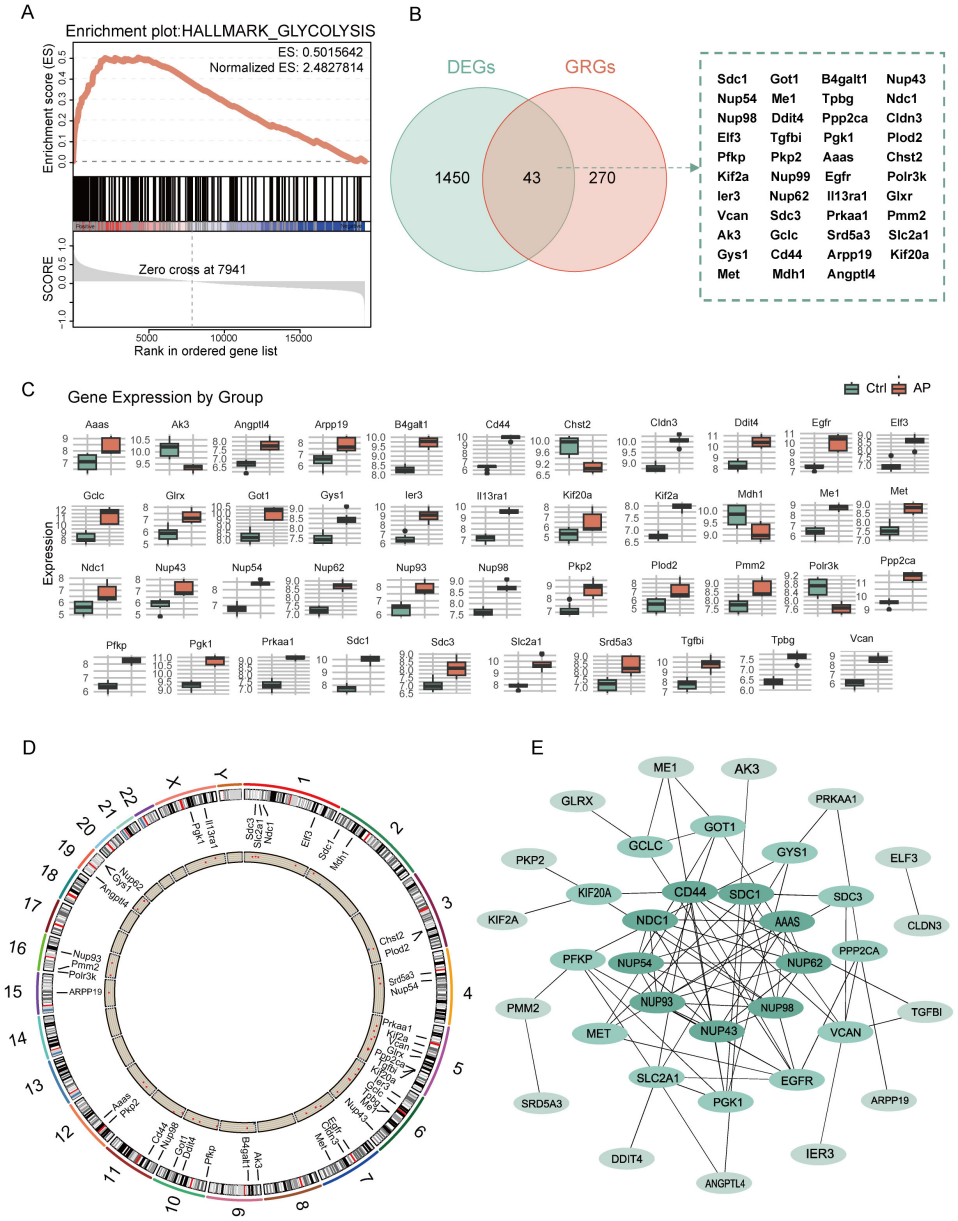
Analysis of glycolysis-related DEGs in AP. **(A)** GSEA enrichment plot showing the significant enrichment of the glycolysis pathway in AP. **(B)** Venn diagram showing the overlap between glycolysis-related genes and DEGs. **(C)** Boxplot depicting the relative expression levels of glycolysis-related DEGs between the control and AP samples. **(D)** Circular plot showing the chromosomal distribution of glycolysis-related DEGs. **(E)** PPI network of glycolysis-related DEGs, with node color intensity representing the MCC score. DEGs, differentially expressed genes; GRGs, glycolysis-related genes; ES, enrichment score; AP, acute pancreatitis; Ctrl, control; MCC, maximal clique centrality.

GSEA revealed significant enrichment of the glycolysis pathway in AP samples, with the NES of 2.48 and FDR below 0.25 (**Figure 3A**). By intersecting 1,483 DEGs with 313 glycolysis-related DEGs using a Venn diagram, 43 glycolysis-related DEGs were identified (**Figure 3B**). Boxplots comparing expression levels of the 43 glycolysis-related DEGs between AP and Ctrl groups showed distinct expression differences (**Figure 3C**). The chromosomal distribution revealed that glycolysis-related DEGs are clustered on chromosomes 5 and 6(**Figure 3D**, **Supplementary Figure 2**). A PPI network of the glycolysis-related DEGs was constructed based on the MCC algorithm to investigate potential interactions (**Figure 3E**, **Supplementary Figure 3**). Node colors represent MCC scores, highlighting hub genes within the network.

### 3.3 Identification of CD44 and CLDN3 as key glycolysis-related DEGs in AP

**Figure 4:**
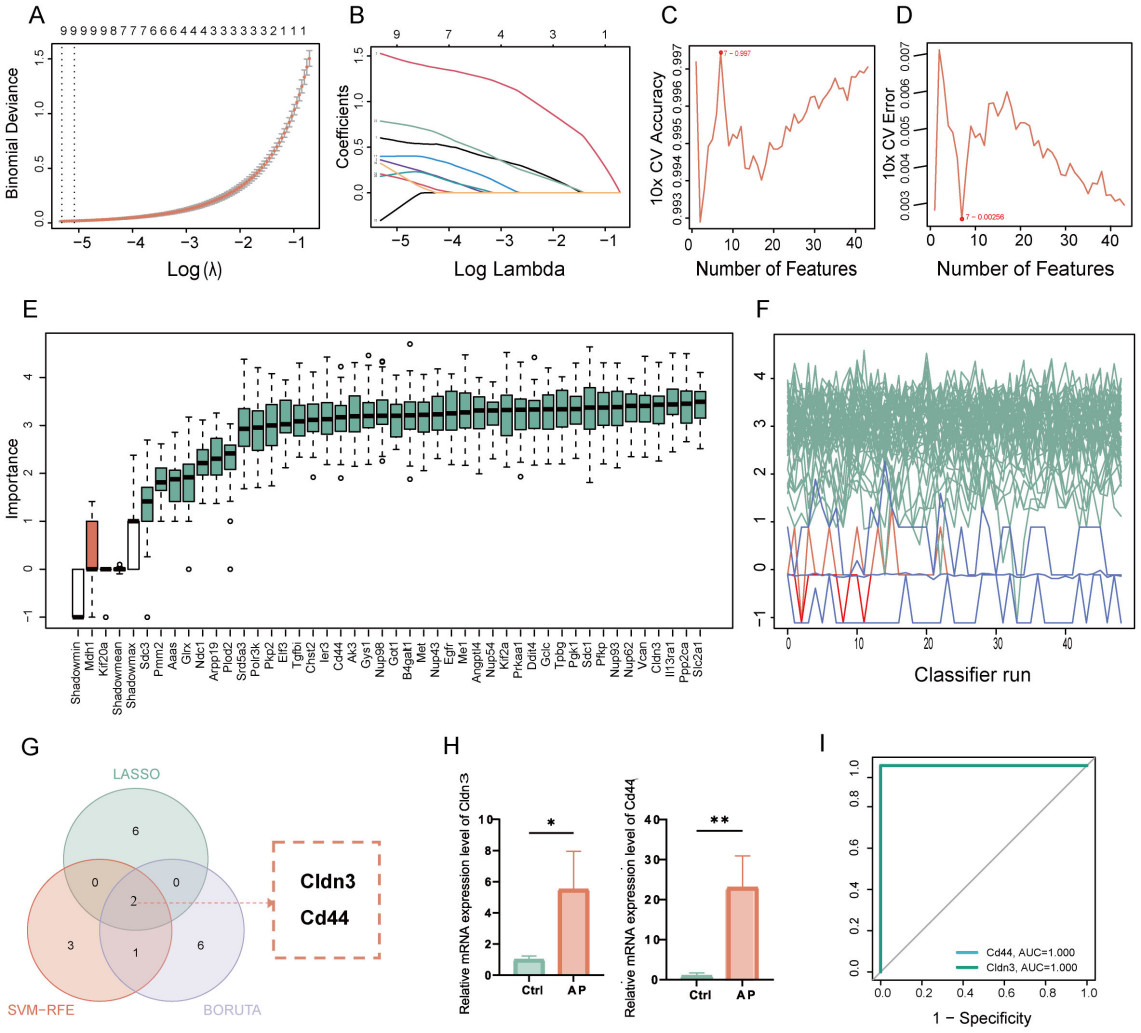
Identification and validation of key glycolysis-related DEGs through machine learning. **(A)** LASSO coefficient paths with vertical dashed lines marking lambda. min and lambda. 1se. **(B)** Mean cross-validation error across lambda values, highlighting the bias-variance trade-off. **(C)** Plot of SVM accuracy rates across different models. **(D)** Plot of SVM error rates across different models. **(E)** Boruta feature importance across iterations, with green for confirmed, red for rejected, and blue representing the minimum, average, and maximum Z-scores of shadow attributes. **(F)** Boruta feature Z-scores, with green for confirmed, red for rejected, and white boxes representing the minimum, average, and maximum Z-scores of shadow attributes. **(G)** Venn diagram showing the overlap of glycolysis-related key genes. **(H)** Relative mRNA expression of the key glycolysis-related DEGs in the validation set. **(I)** ROC curve of the glycolysis-related genes in the GSE169076 validation set. Ctrl, control; AP, acute pancreatitis. *p≤0.05,**p≤0.01.

Glycolysis-related key DEGs of AP were identified by utilizing machine learning approaches. LASSO regression selected key features with optimal lambda values determined by cross-validation (**Figures 4A, B**). SVM-RFE models were evaluated through accuracy and error rate analyses across multiple parameters (**Figures 4C, D**). Boruta feature selection further refined candidates by assessing feature importance and Z-scores over multiple iterations (**Figures 4E, F**). Integrating results from these methods identified 2 overlapping key glycolysis-related DEGs: Claudin-3 (CLDN3) and CD44 molecule (**Figure 4G**). Validation in an independent dataset confirmed significant upregulation of these genes in the AP samples (**Figure 4H**). In the validation datasets, ROC analysis yielded an Area Under the Curve (AUC) of 1.0 for both Cldn3 and Cd44 (**Figure 4I**, **Supplementary Figure 4**). Rather than indicating diagnostic applicability, these results primarily serve as evidence that the differential expression of these genes is robust and consistently observed across independent datasets.

### 3.4 GSEA on GO terms of CD44 and CLDN3

**Figure 5:**
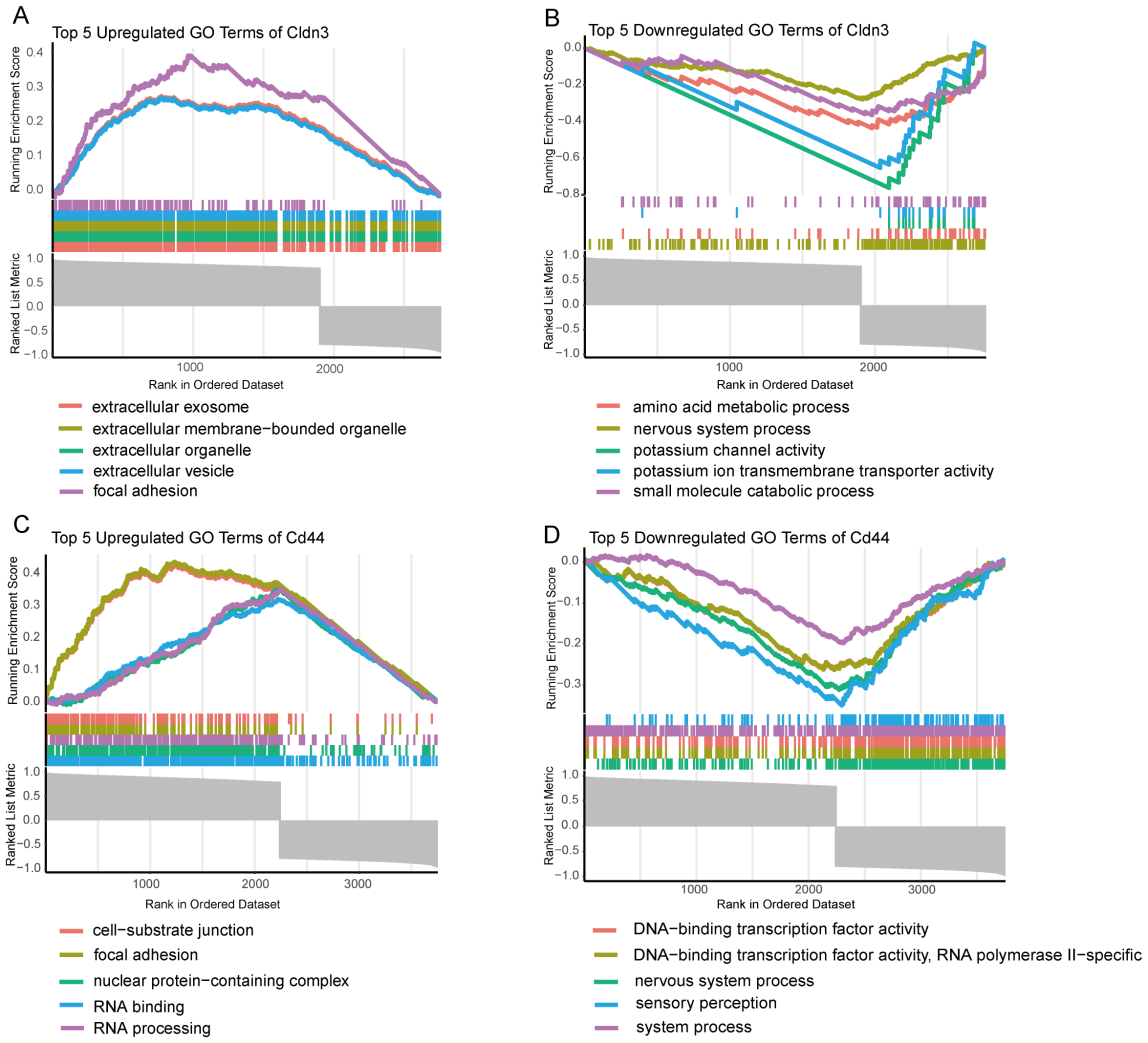
GSEA enrichment analysis of GO terms for key Genes. **(A)** Top 5 upregulated GO terms for CLDN3 based on GSEA analysis. **(B)** Top 5 downregulated GO terms for CLDN3 based on GSEA analysis. **(C)** Top 5 upregulated GO terms for CD44 based on GSEA analysis. **(D)** Top 5 downregulated GO terms for CD44 based on GSEA analysis.

GSEA was performed on GO terms to explore the biological roles of CLDN3 and CD44 (**Figure 5**). The upregulated GO terms of these two genes were related to cell adhesion, extracellular vesicle formation, and RNA processing, which were essential for immune cell communication, migration, and activation. Conversely, the downregulated GO terms were enriched in sensory perception, ion transport, and transcription factor activity, which reflected a suppression of neuronal-like signaling pathways. These results suggest that CLDN3 and CD44 may contribute to immune-mediated structural remodeling and transcriptional regulation within the inflammatory microenvironment of AP.

### 3.5 Single-cell transcriptomic profile of CD44 and CLDN3

**Figure 6:**
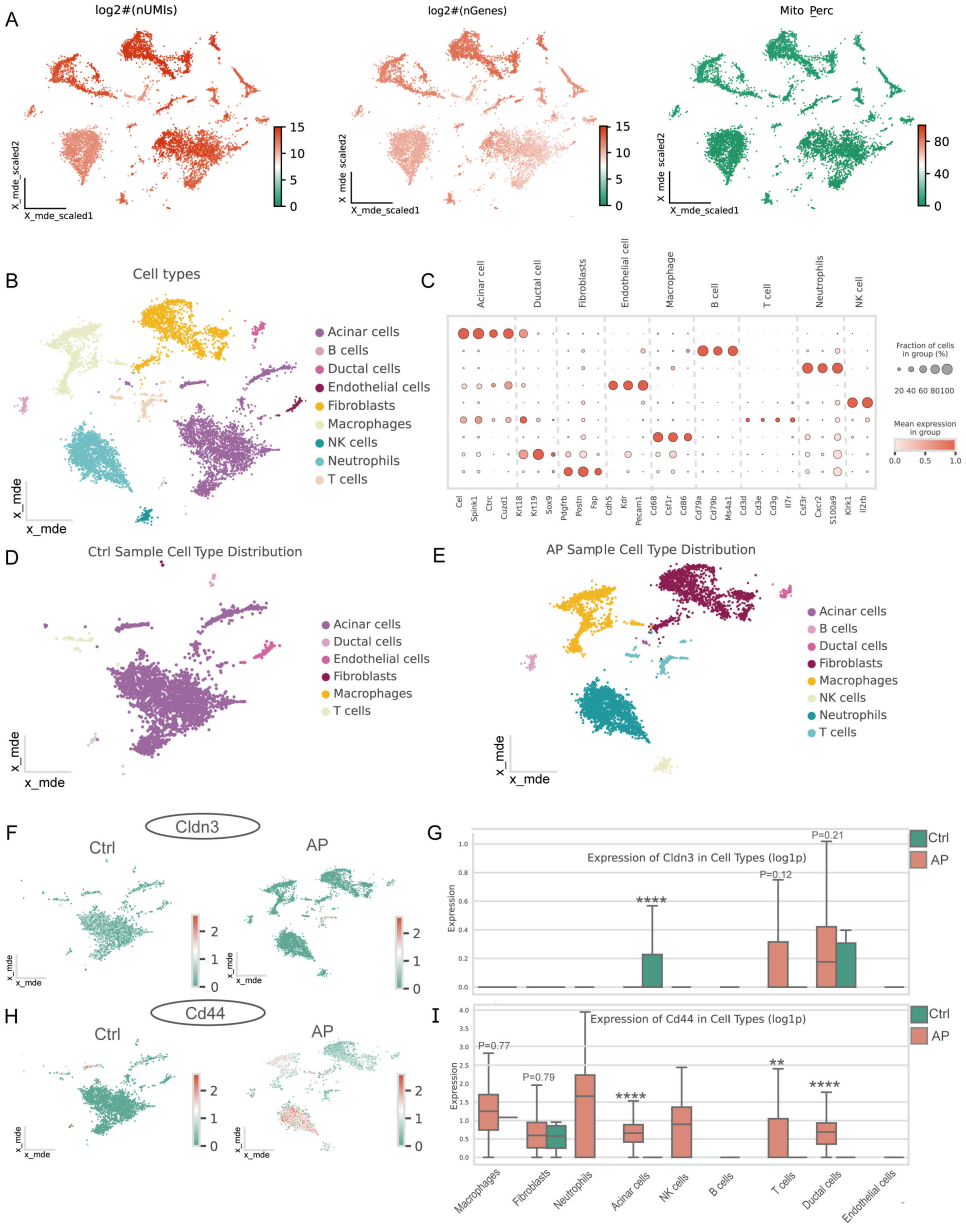
Single-cell characteristics of the pancreas in AP and features of glycolysis-related key DEGs. **(A)** Distribution of log-transformed UMI counts, gene counts, and mitochondrial gene expression percentage across cells, providing insights into the cellular quality of the dataset. **(B)** Major cell types in the pancreas. **(C)** Marker genes for different cell types. **(D)** Major cell types in the pancreas of the AP mice model. **(E)** Major cell types in the pancreas of healthy mice. **(F, H)** Spatial distribution of CLDN3 and CD44 in the pancreas from control and AP mice within the single-cell atlas. **(G, I)** Expression of CLDN3 and CD44 across major pancreatic cell types between AP and control.AP, acute pancreatitis; Ctrl, control. **p ≤ 0.01, ****p ≤ 0.0001.

Single-cell quality control confirmed high data integrity (**Figure 6A**). Unsupervised clustering and marker-gene annotation identified the main cell populations of the pancreas—acinar, ductal, endothelial, fibroblast, B-cell, T-cell, NK-cell, macrophage, and neutrophil clusters (**Figures 6B, C**). There was an increasing number of immune cell populations and a remarkable loss of acinar cells in AP samples (**Figures 6D, E**). Cell-type–resolved mapping revealed the expression changes of glycolysis-related key genes in AP (**Figures 6F-I**). CD44 was enriched in both ductal and immune cells, which is consistent with the enhanced activation and migration of the immune cells. Notably, CLDN3 was significantly downregulated in acinar cells, indicating a disruption in epithelial integrity. This finding was inconsistent with bulk RNA-seq results, thereby prompting subsequent protein-level validation.

### 3.6 Immune infiltration and correlation analysis of CD44 and CLDN3 on ssGSEA

**Figure 7:**
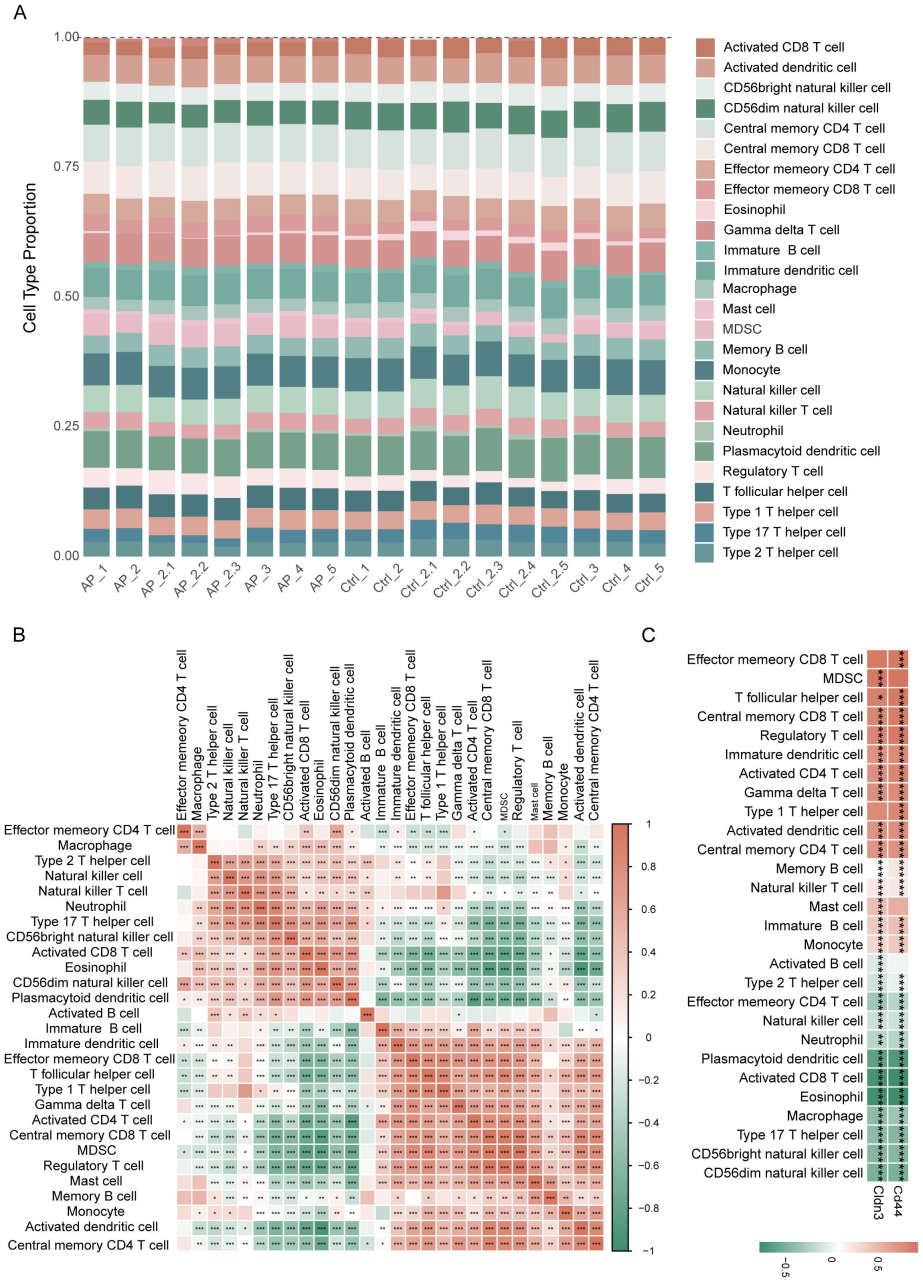
Immune infiltration analysis based on ssGSEA. **(A)** Proportions of immune cell types among different samples. **(B)** Heatmap depicting the correlation analysis of immune cells. **(C)** Heatmap showing the relationship between glycolysis-related key DEGs and immune cell infiltration. *p≤0.05,**p≤0.01,***p≤0.001.

The ssGSEA analysis revealed distinct immune infiltration patterns of the pancreas in AP (**Figure 7A**). An increase in the expression of immune cell subsets was observed, indicating an alteration from a steady-state immune surveillance in normal status to an activated state. Correlation analysis among immune cells demonstrated the coordinated infiltration patterns, which indicated synergistic immune responses to the microenvironment in AP (**Figure 7B**). Notably, glycolysis-related key DEGs showed strong correlations with the majority of immune cells (**Figure 7C**, **Supplementary Table 5**), suggesting their broad regulatory roles in shaping the immune landscape during AP.

### 3.7 Prediction of TFs and miRNA of CD44 and CLDN3

**Figure 8:**
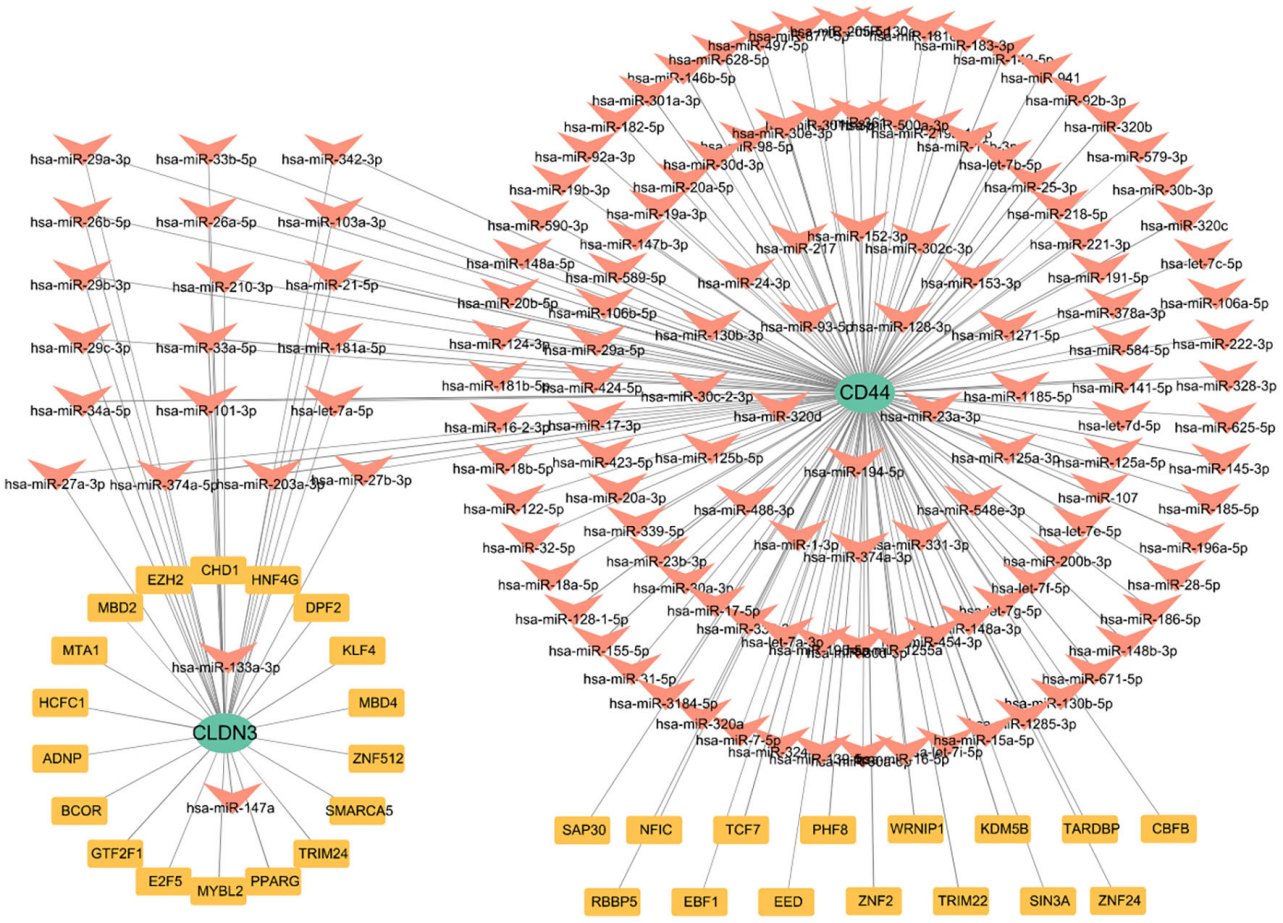
The TFs and miRNA regulatory network of CLDN3 and CD44.

To elucidate the upstream regulation of the glycolysis-related key DEGs, TFs, and miRNA networks were assembled from curated datasets (**Figure 8**). A total of 33 TFs were predicted to target these genes. At the post-transcriptional level, 138 miRNAs were identified to regulate the glycolysis-related key DEGs. Focusing on miRNAs with a degree of ≥1, 21 miRNAs were shared between CD44 and CLDN3(**Supplementary Figure 6**). Notably, SAP30, KDM5B, and PHF8 were identified as TFs regulating CD44, while TRIM24, SMARCA5, E2F5, and BCOR were predicted to regulate CLDN3. The identification of these shared miRNAs suggests post-transcriptional regulation of CD44 and CLDN3.

### 3.8 Validation of CD44 and CLDN3 in experimental AP model

**Figure 9:**
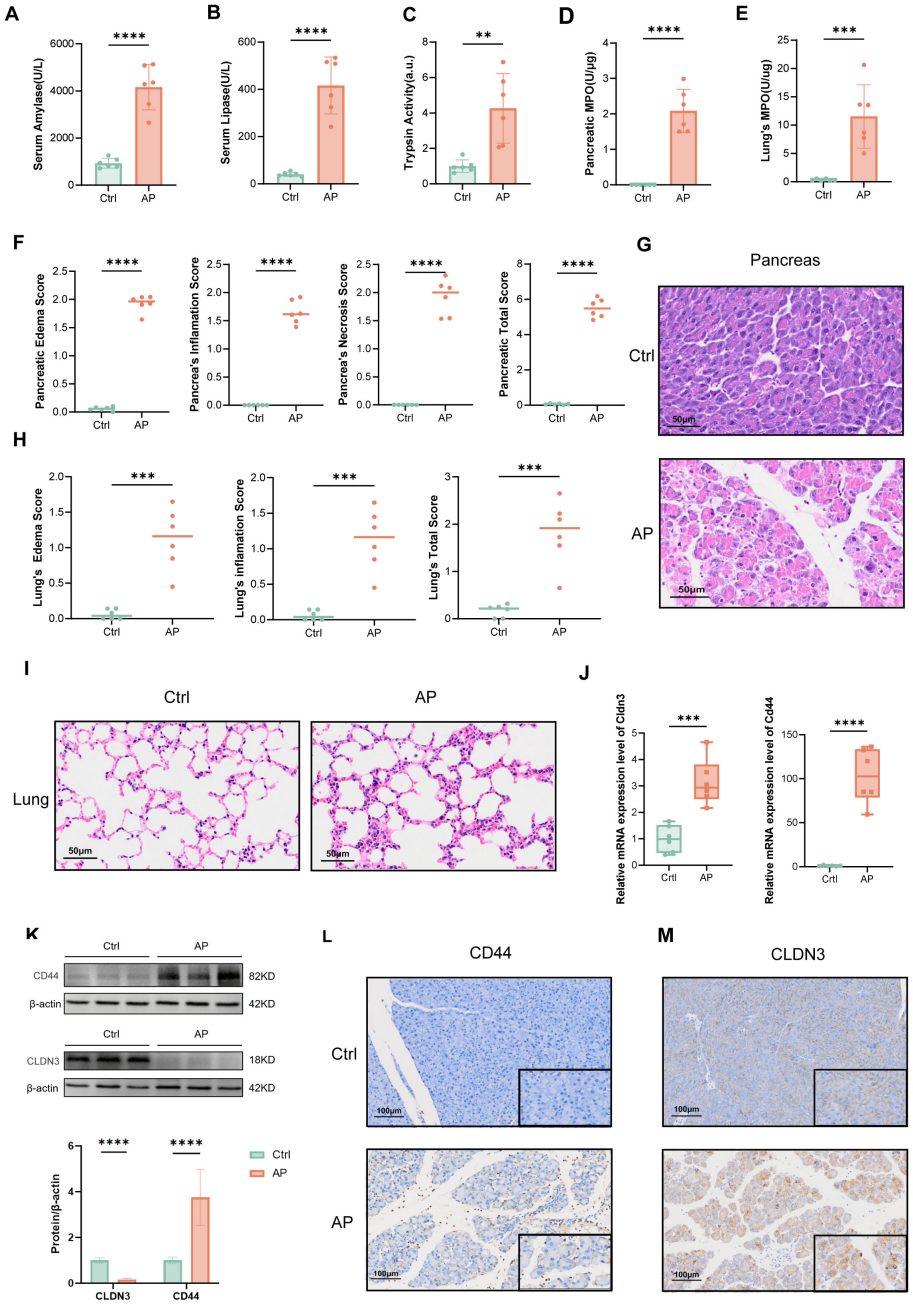
Validation of the AP model and expression of CLDN3 and CD44. (n = 6 biologically independent samples). Serum amylase levels. **(B)** Serum lipase levels. **(C)** Trypsin activity. **(D)** MPO activity in pancreatic tissue. **(E)** MPO activity in lung tissue. **(F)** Quantitative histopathological scores of the pancreas. **(G)** Representative H&E-stained images of pancreatic tissue from control and AP mice (200x). **(H)** Histopathological scores of the lung. **(I)** Representative H&E-stained images of lung tissue from control and AP mice (200x). **(J)** Relative mRNA expression levels of Cldn3 and Cd44 in pancreatic tissues. **(K)** Western blot analysis of CD44 and CLDN3 with quantification of band intensity normalized to β-actin. **(L, M)** IHC staining and semi-quantitative analysis of CLDN3 and CD44 in pancreatic tissues from control and AP mice (100x,400x). MPO, myeloperoxidase. **p≤0.01,***p≤0.001,****p≤0.0001.

To validate the bioinformatic findings, we established a mouse AP model by the combination of caerulein and LPS, as confirmed by serum amylase, lipase, and trypsin activity, and MPO activity in pancreatic tissue (**Figures 9A-D**), as well as H&E histopathological assessment (**Figures 9F, G**). The elevation of lung MPO activity and H&E scores indicated the involvement of the lungs (**Figure 9E, H, I**). We next assessed Cd44 and Cldn3 expression at both mRNA and protein levels by qPCR and western blotting (**Figures 9J, K**). Cd44 was upregulated at both transcriptional and protein levels in AP mice. Correspondingly, IHC staining showed that CD44 was predominantly expressed in inflammatory cells within pancreatic tissues of AP mice (**Figure 9L**). Interestingly, bulk qPCR indicated increased Cldn3 mRNA, whereas Western blotting revealed a marked protein reduction. This discrepancy likely reflects cell-type compositional shifts in AP, whereby increased Cldn3 expression in infiltrating immune and ductal cells masks its downregulation in acinar cells(p<0.0001), as revealed by single-cell transcriptomics (**Figure 6G**). IHC further showed a redistribution of CLDN3 from acinar membranes in controls to diffuse cytoplasmic staining in AP mice(**Figure 9M**).

## 4 Discussion

To the best of our knowledge, this is the first study to investigate glycolysis and its immunoregulatory role in AP by integrating bulk and single-cell transcriptomic analyses, machine learning, and experimental validation. The results identify new insights of CD44 and CLDN3 into glycolysis-driven metabolic-immune mechanisms, and provide potential biomarkers and therapeutic targets for AP.

The cellular landscape in immunoregulation in AP is systematically constructed. Upregulation of glycolysis-related genes is consistent with immune and inflammatory mediators, highlighting the metabolic-immune interaction relationship in AP. Functional enrichment analyses of the DEGs and single-cell analysis reveal a complex interaction of immune cells infiltration and the loss of pancreatic acinar cells in AP, indicating tissue injury and immune activation during disease progression. Two immune cell clusters are shown in AP immune landscape: one cluster includes memory T cells, dendritic cells, and regulatory cells, which reflect a microenvironment in alleviating inflammation by immune regulation and suppression; the other cluster is consisted of activated B cells, natural killer cells, and effector cells, which represent a state of upregulated inflammatory response and innate immune activation. These findings underscore the complex immune regulatory mechanisms of the immune microenvironment in AP and suggest the potential of distinct immune cells as therapeutic strategies for AP. Significant enrichment of glycolytic pathways in AP tissues is confirmed by GSEA analysis. Key adhesion molecules and nuclear pore complex components are identified as central hubs, indicating the critical roles in immune cell adhesion and nuclear transport. Notably, CD44 and CLDN3 are identified via a combination of deep learning algorithms and validated in the experimental AP model.

CD44 is a cell surface adhesion molecule that plays extensive roles in the activation, recirculation, and homing of immune cells (35–39). It has been reported as a biomarker of cancer stem cells and facilitates metastasis, immune evasion, and therapeutic resistance (40–42). Recent studies demonstrate the role of CD44 in metabolic regulation, particularly in shifting glycolysis and oxidative phosphorylation (OXPHOS). CD44 modulates glycolytic metabolism by regulating key enzymes LDHA, LDHB, and PFKFB4 via HIF-1α and AMPK/mTOR signaling (43–47). In our study, CD44 is enriched in AP samples within pathways related to cell–matrix adhesion and RNA processing. In contrast, pathways associated with sensory perception and transcription factor activity are downregulated. These findings suggest that CD44 may contribute to immune cell infiltration, post-transcriptional regulation, and cellular adaptation to inflammatory stress in AP. We identify CD44 as a crucial glycolysis-related DEG and validate its upregulated expression among a series of cells, including neutrophils, acinar cells, and ductal epithelial cells in AP samples. Correlation analysis between CLDN3 and CD44 with immune cell infiltration reveals significant associations with multiple immune cell types. These findings suggest that glycolysis–related key genes not only contribute to metabolic reprogramming but may also modulate immune cell infiltration, thereby influencing the immune microenvironment and pathological progression of AP.

CLDN3, which is highly expressed in epithelial and endothelial tissues (48, 49), is a crucial tight junction protein for maintaining epithelial barrier integrity. The dysregulation of CLDN3 has been implicated in cancer (50–55) and inflammatory diseases (56–58). Recent evidence (59–63) further suggests that CLDN3 expression is altered with elevated glycolytic activity in metabolic stress and tumors. In this study, we identified a discrepancy between the elevation of Cldn3 mRNA in bulk transcriptomic/qPCR data and the reduction of protein-level measurements by western blotting. Our ScRNA-seq transcriptomic data can help to resolve this paradox by revealing marked Cldn3 downregulation in acinar cells, alongside upregulation in ductal cells and T cells. During AP, massive immune cell infiltration and possible ductal hyperplasia can elevate the bulk mRNA signals despite acinar-specific suppression. However, because non-acinar cells contribute minimally to the total pancreatic protein pool, and inflammatory stress may promote CLDN3 internalization and degradation, the overall protein abundance still declines. IHC further revealed CLDN3 redistribution from the apical membrane to the cytoplasm in AP, a pattern consistent with tight junction disassembly and loss of epithelial polarity (64). Inflammatory cytokines such as TNF-α can trigger internalization of tight junction proteins, causing their removal from the membrane and cytoplasmic accumulation (65). In addition, disruption of polarity complexes such as PAR3/aPKC can facilitate endocytic uptake and lysosomal degradation of tight junction components (64, 66, 67). These inflammation-driven processes provide a mechanistic basis for the CLDN3 relocalization observed in AP. Collectively, these findings suggest that CLDN3, beyond its classical barrier role, participates in epithelial remodeling and immune modulation during AP.

Upstream regulators of glycolysis–related key genes are studied by constructing the TFs–mRNA–miRNA regulatory network. SAP30, KDM5B, and PHF8 are primarily epigenetic modifiers that may regulate CD44 expression via epigenetic regulation. TRIM24, SMARCA5, E2F5, and BCOR potentially regulate CLDN3 expression as transcriptional regulators and chromatin remodelers. Additionally, 21 shared miRNAs were computationally identified that may be involved in the post-transcriptional regulation of both CD44 and CLDN3, potentially linking these genes to immune modulation and metabolic processes in AP. These findings suggest a possible multilayered regulatory mechanism governing key glycolysis-related genes in AP.

This study explores the role of glycolysis in immune regulation in AP by using bulk transcriptomic, ScRNA-seq data, and experimental validation. However, there are several limitations. Firstly, it is limited by the exclusive use of a homogeneous mouse model and the absence of human patient data, which may introduce confounding factors and restrict the direct translational relevance of our findings. Future studies need to integrate the data from multiple animal models and clinical samples to validate the generalizability and clinical applicability of these observations. Secondly, the validation datasets yielded perfect AUC values for Cd44 and Cldn3. This phenomenon likely arises from intrinsic features of animal models, including controlled experimental conditions, synchronized sample collection, uniform disease induction, and inflammation-driven transcriptional changes, all of which can exaggerate group differences. Future validation in diverse clinical cohorts remains essential. Thirdly, although we systemically use GSEA, ScRNA-seq analysis, immune infiltration, and TFs-mRNA-miRNA analyses to explore potential regulatory mechanisms, this study remains preliminary. In-depth mechanistic studies are needed to elucidate the molecular mechanisms of glycolysis in regulating immune responses in AP.

## 5 Conclusion

This study identifies that CD44 and CLDN3 play crucial roles in metabolic regulation and immune modulation in AP, offering novel insights for biomarkers and therapeutic targets of the disease. Future researches are required to investigate the mechanisms underlying glycolysis-immune interactions of these genes in AP.

## Data Availability

The original contributions presented in the study are included in the article/**Supplementary Material**. Further inquiries can be directed to the corresponding author.
