# Supplementary material for: CD44 and CLDN3 as immune-metabolic regulators in acute pancreatitis: a multi-modal transcriptomics study and experimental validation
Source: Front Immunol. 2025 Oct 22;16:1665200. doi: 10.3389/fimmu.2025.1665200 (PMC12586124; doi:10.3389/fimmu.2025.1665200)
Supplement: Supplementary file 1 [file DataSheet1.pdf]

Supplementary Material

1 Supplementary Data

1.1 Reagents and method

1.1.1 Western Blot

Total protein was extracted from pancreatic tissues using strong RIPA lysis buffer (Beyotime Biotechnology, China; Cat. No. P0013B), supplemented with protease inhibitors (Sigma – Aldrich/Roche, USA; Cat. No. 4693159001) and phosphatase inhibitors (Sigma – Aldrich/Roche, USA; Cat. No. 04906837001). Protein concentration was determined using the Bradford Protein Assay Kit (Thermo Fisher Scientific; Cat. No ZA382177). Protein samples were separated by SDS-PAGE and transferred onto 0.45 μm PVDF membranes (Merck Millipore Ltd, Germany; Cat. No IPVH00010). Membranes were blocked for 1 hour at room temperature (Bio-Rad Laboratories, USA; Cat. No. 1706404), then incubated overnight at 4°C with the following primary antibodies: anti-CLDN3 (1:1500, Abcam, ab317319), anti-CD44 (1:2000, Abcam, ab189524), and anti-β-actin (1:20000, Proteintech, 66009-1-Ig). After washing with TBST, membranes were incubated with HRP-conjugated goat anti-mouse IgG (1:20000, HUABIO, China; HA1006) or HRP-conjugated goat anti-rabbit IgG antibody (1:50000, HUABIO, China; HA1001) for 1 hour at room temperature. Signals were visualized using the ECL detection system (Beijing 4A Biotech Co., Ltd, China; Cat. No. 4AW011-100), and band intensity was quantified using ImageJ software.

1.1.2 Immunohistochemistry

Paraffin-embedded pancreatic tissues were sectioned (4 μm) and subjected to standard IHC procedures, including dewaxing, rehydration, antigen retrieval, blocking of endogenous peroxidase with 3% H_2O_2, and serum blocking. Sections were incubated overnight at 4 °C with the following primary antibodies: anti-CLDN3 (1:2000, Abcam, ab317319) and anti-CD44 (1:4000, Abcam, ab189524). After washing with PBS, slides were incubated with HRP-conjugated goat anti-rabbit secondary antibody (1:200, Servicebio, GB23303) for 50 min at room temperature. Visualization was achieved using a DAB detection kit, and nuclei were counterstained with hematoxylin. Finally, slides were dehydrated, cleared in xylene, and mounted with neutral resin. Positive staining appeared as brown-yellow in the cytoplasmic or membranous regions.

2 Supplementary Figures and Tables

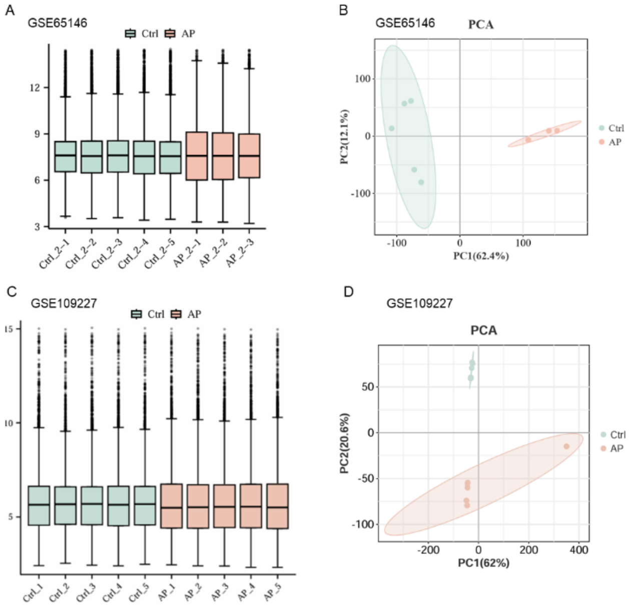

Supplementary Figure 1. Quality control and sample distribution analysis of the datasets. (A, C). Boxplots showing the distribution of gene expression values across all samples in the GSE65146 and GSE109227 datasets, respectively, demonstrating effective normalization and overall consistency. (B, D). Principal Component Analysis plot illustrating the clustering pattern among samples, reflecting potential group separation or underlying biological variability.

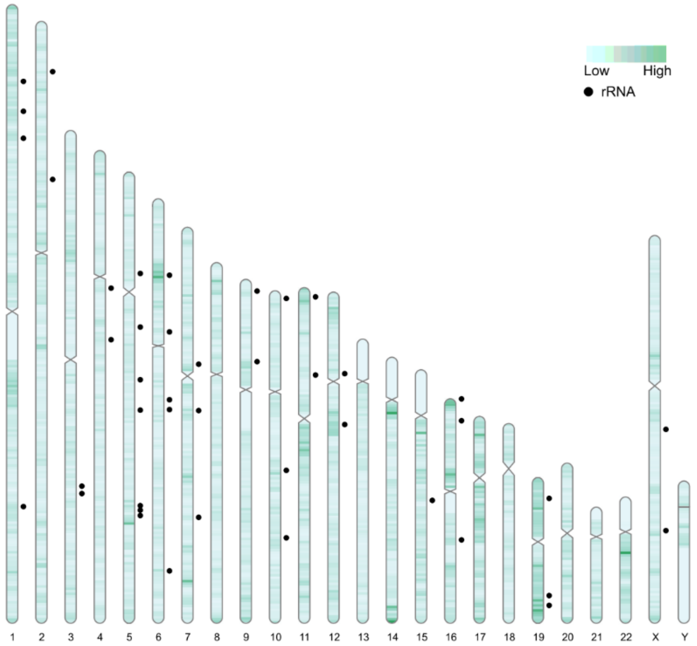

Supplementary Figure 2. Chromosomal distribution of glycolysis-related DEGs.

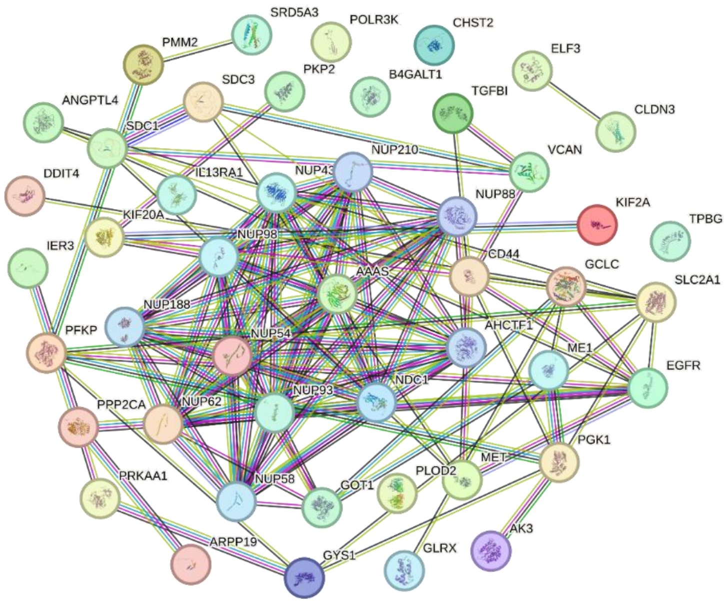

Supplementary Figure 3. PPI network of glycolysis-related DEGs.

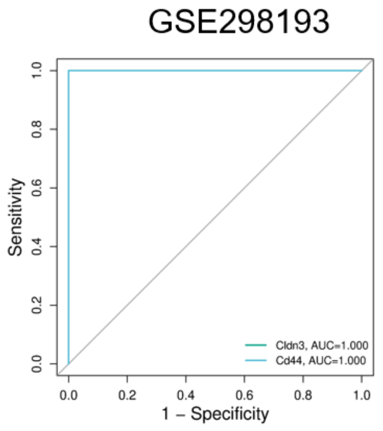

Supplementary Figure 4. ROC curve of the glycolysis-related genes in the GSE298193 validation set.

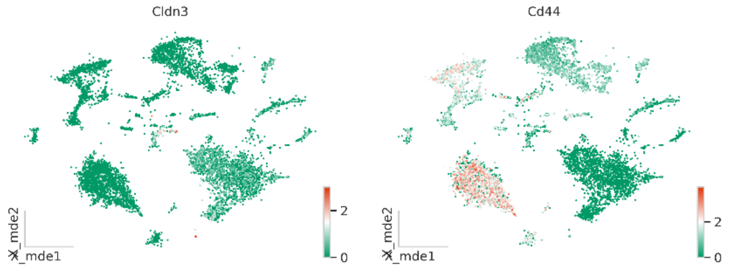

Supplementary Figure 5. Single-cell distribution map of CLDN3 and CD44 in pancreatic tissue.

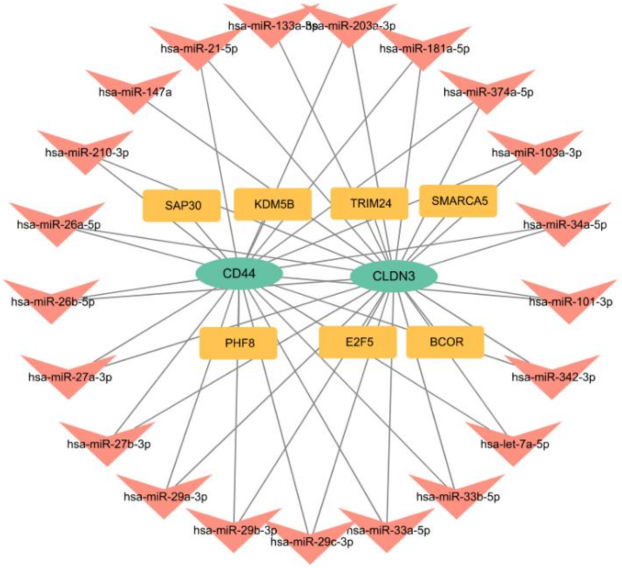

Supplementary Figure 6. The TFs and miRNA regulatory network of CLDN3 and CD44 (degree > 1).

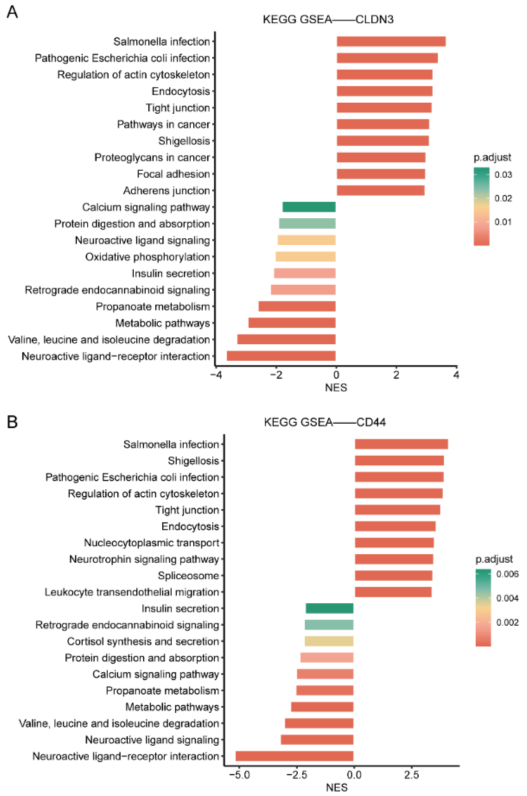

Supplementary Figure 7. Bar plot of KEGG pathway enrichment analysis for CLDN3 and CD44 based on GSEA.

Supplementary Table 1. Datasets

Dataset	Platform	Source	Species	Samples (Ctrl)	Samples (AP)	Attribute
GSE109227	GPL6246	Pancreatic tissue	Mouse	5	5	Training set
GSE65146	GPL6246	Pancreatic tissue	Mouse	5	3	Training set
GSE169076	GPL23479	Pancreatic tissue	Mouse	3	3	Validation set
GSE298193	GPL25947	Pancreatic tissue	Mouse	3	3	Validation set
GSE279876	GPL19057	Pancreatic tissue	Mouse	1	1	Single-cell set

Supplementary Table 2. Glycolysis-related gene sets

No.	Glycolysis-related gene sets
1	WP_GLYCOLYSIS_IN_SENESCENCE.v20
2	WP_AEROBIC_GLYCOLYSIS.v2024.1.H
3	MODULE_306.v2024.1.Hs
4	KEGG_GLYCOLYSIS_GLUCONEOGENESIS
5	HALLMARK_GLYCOLYSIS.v2024.1.Hs
6	REACTOME_GLYCOLYSIS.v2024.1.Hs

7	BIOCARTA_GLYCOLYSIS_PATHWAY.v20
8	WP_GLYCOLYSIS_AND_GLUCONEOGENES
9	WP_AEROBIC_GLYCOLYSIS_AUGMENTED

Supplementary Table 3. Gene-specific primers for qRT-PCR

Gene	Forward(5'-3')	Reverse(5'-3')
Cldn3	GCAAGGACTACGTCTGAGGG	ACTGTGTGTCGTCTGTCACC
Cd44	CACCATTGCCTCAACTGTGC	TTGTGGGCTCCTGAGTCTGA

Supplementary Table 4. Immune cell markers

**Immune cell type**	**Marker Genes**
Activated B cell	ADAM28, CD180, CD79B, BLK, CD19, MS4A1, TNFRSF17, IGHM, GNG7, MICAL3, SPIB, HLA-DOB, IGKC, PNOC, FCRL2, BACH2, CR2, TCL1A, AKNA, ARHGAP25, CCL21, CD27, CD38, CLEC17A, CLEC9A, CLECL1
Activated CD4 T cell	AIM2, BIRC3, BRIP1, CCL20, CCL4, CCL5, CCNB1, CCR7, DUSP2, ESCO2, ETS1, EXO1, EXOC6, IARS, ITK, KIF11, KNTC1, NUF2, PRC1, PSAT1, RGS1, RTKN2, SAMSN1, SELL, TRAT1
Activated CD8 T cell	C1GALT1C1, CCT6B, CD37, CD3D, CD3E, CD3G, CD69, CD8A, CETN3, CSE1L, GEMIN6, GNLY, GPT2, GZMA, GZMH, GZMK, IL2RB, LCK, MPZL1, NKG7, PIK3IP1, PTRH2, TIMM13, ZAP70
Central memory CD4 T cell	ABHD3, AHNAK, ANXA2P2, AQP3, ATHL1, BMI1, BZW2, CD63, COL4A1, CYLD, ELMO2, FYN, GLIPR1, GSS, IFITM2, ITGB1, ITGB2, KLF5, LSP1, NDUFB9, PKM2, SFXN3, SIRPG, SMAD4, STX4, TRADD, VIM, XRCC6

Central memory CD8 T cell	ADAM12, ADCY9, F13A1, FCER1G, FCGR3B, FGF7, FKBP4, GLUD1, GM2A, GUSB, IL1RN, NOL11, NTRK1, RARA, RNF128, SIGLEC1, TNFRSF11A, TOX4, UBA52, ULBP1
Effector memory CD4 T cell	ATM, CASP3, CASQ1, CD300E, DARS, DOCK9, EXOSC9, EZH2, GDE1, IL34, NCOA4, NEFL, PDGFRL, PTGS1, REPS1, SCG2, SDPR, SIGLEC14, SIGLEC6, TAL1, TFEC, TIPIN, TPK1, UQCRB, USP9Y, WIPF1, ZCRB1
Effector memory CD8 T cell	ACAP1, APOL3, ARHGAP10, ATP10D, C3AR1, CCR5, CD160, CD55, CFLAR, CMKLR1, DAPP1, FCRL6, FLT3LG, GZMM, HAPLN3, HLA-DMB, HLA-DPA1, HLA-DPB1, IFI16, LIME1, LTK, NFKBIA, SETD7, SIK1, TRIB2
Gamma delta T cell	ACP5, AQP9, BTN3A2, C1orf54, CARD8, CCL18, CD209, CD33, CD36, CDK5, IL10RB, KLRF1, LGALS1, MAPK7, KLHL7, KRT80, LAMC1, LCORL, LMNB1, MEIS3P1, MPL, FABP1, FABP5, FADD, MFAP3L, MINPP1, RPS24, RPS7, RPS9, DBNL, CCL13
Immature B cell	CD22, CYBB, FAM129C, FCRL1, FCRL3, FCRL5, FCRLA, HDAC9, HLA-DQA1, HVCN1, KIAA0226, NCF1, NCF1B, P2RY10, SP100, TXNIP, STAP1, TAGAP, ZCCHC2
Memory B cell	AICDA, CCNA2, CDKN3, CLCN5, ENPP1, FCER1A, FCRL4, MYC, RUNX2, SORL1, SOX5, STAT5A, STAT5B, TLR9
Regulatory T cell	CCL3L1, CD72, CLEC5A, FOXP3, ITGA4, L1CAM, LIPA, LRP1, LRRC42, MARCO, MMP12, MNDA, MRC1, MS4A6A, PELO, PLEK, PRSS23, PTGIR, ST8SIA4, STAB1
T follicular helper cell	B3GAT1, CDK5R1, PDCD1, BCL6, CD200, CD83, CD84, FGF2, GPR18, CEBPA, CECR1, CLEC10A, CLEC4A, CSF1R, CTSS, DMN, DPP4, LRRC32, MC5R, MICA, NCAM1, NCR2, NRP1, PDCD1LG2, PDCD6, PRDX1, RAE1, RAET1E, SIGLEC7, SIGLEC9, TYRO3, CHST12, CLIC3, IVNS1ABP, KIR2DL2, LGMN
Type 1 T helper cell	CD70, TBX21, ADAM8, AHCYL2, ALCAM, B3GALNT1, BBS12, BST1, CD151, CD47, CD48, CD52, CD53, CD59, CD6, CD68, CD7, CD96, CFHR3, CHRM3, CLEC7A, COL23A1, COL4A4, COL5A3, DAB1, DLEU7, DOC2B, EMP1, F12, FURIN, GAB3, GATM, GFPT2, GPR25, GREM2, HAVCR1, HSD11B1, HUNK, IGF2, RCSD1, RYR1, SAV1, SELE, SELP, SH3KBP1, SIT1, SLC35B3, SIGLEC10, SKAP1, THUMPD2, TIGIT, ZEB2, ENC1, FAM134B, FBXO30, FCGR2C, STAC, LTC4S, MAN1B1, MDH1, MMD, RGS16, IL12A, P2RX5, CD97, ITGB4, ICAM3, METRNL, TNFRSF1A, IRF1, HTR2B, CALD1, MOCOS, TRAF3IP2, TLR8, TRAF1, DUSP14

Type 17 T helper cell	IL17A, IL17RA, C2CD4A, C2CD4B, CA2, CCDC65, CEACAM3, IL17C, IL17F, IL17RC, IL17RE, IL23A, ILDR1, LONRF3, SH2D6, TNIP2, ABCA1, ABCB1, ADAMTS12, ANK1, ANKRD22, B3GALT2, CAMTA1, CCR9, CD40, GPR44, IFT80
Type 2 T helper cell	ASB2, CSRP2, DAPK1, DLC1, DNAJC12, DUSP6, GNAI1, LAMP3, NRP2, OSBPL1A, PDE4B, PHLDA1, PLA2G4A, RAB27B, RBMS3, RNF125, TMPRSS3, GATA3, BIRC5, CDC25C, CDC7, CENPF, CXCR6, DHFR, EVI5, GSTA4, HELLS, IL26, LAIR2
Activated dendritic cell	ABCD1, C1QC, CAPG, CCL3L3, CD207, CD302, ATP5B, ATP5L, ATP6V1A, BCL2L1, C1QB, SNURF, SPCS3, CCNA1, CEACAM8, NOS2, SRA1, TNFRSF6B, TREM1, TREML1, RHOA, SLC25A37, TNFSF14, TREML4, VNN2, XPO6, CLEC4C, TNFAIP2, UBD, ACTR3, RAB1A, SLA, HLA-DQA2, SIGLEC5, SLAMF9
CD56bright natural killer cell	ABAT, C11orf75, C5orf15, CDHR1, DCAF12, DYNLL1, GPR137B, HCP5, HDGFRP2, KRT86, MLST8, ELMOD3, ENTPD5, FAM119A, FAM179A, CLIC2, COX7A2L, CREB3L4, CSF1, CSNK2A2, CSTA, CSTB, CTPS, CTSD, FST, GATA2, GMPR, HDC, HEY1, HOXA1, HS2ST1, HS3ST1, BCL11B, CDH3, MYL6B, NAA16, ClQA, ClQB, CYP27B1, EIF3M
CD56dim natural killer cell	CYP27A1, DDX55, DYRK2, RPL37A, NOTCH3, AKR7A3, GPRC5C, GRIN1, HLA-E, PORCN, PSMC4, UPP1, IL21R, KIR2DS1, KIR2DS2, KIR2DS5
Eosinophil	GIPR, KRT18P50, LRMP, FOSB, RRP12, GPR183, NR4A3, ST3GAL6, DEPDC5, PDE6C, PKD2L2, GPR65, IL5RA, P2RY14, DACH1, DAPK2, EMR3
Immature dendritic cell	ACADM, AHCYL1, ALDH1A2, ALDH3A2, ALDH9A1, ALOX15, AMT, ARL1, ATIC, ATP5A1, CAPZA1, LILRA5, RDX, RRAGD, TACSTD2, INPP5F, RAB38, PLAU, CSF3R, SLC18A2, AMPD2, CLTB, C1orf162
Macrophage	AIF1, CCL1, CCL14, CCL23, CCL26, CD300LB, CNR1, CNR2, EIF1, EIF4A1, FPR1, FPR2, FRAT2, GPR27, GPR77, RNASE2, MS4A2, BASP1, IGSF6, HK3, VNN1, FES, NPL, FZD2, FAM198B, HNMT, SLC15A3, CD4, TXNDC3, FRMD4A, CRYBB1, HRH1, WNT5B
Mast cell	ADAMTS3, CPA3, CMA1, CTSG, ARHGAP15, CPM, FCN1, FTL, HSPA6, ITGA9, RNASE3, S100A4, SIGLEC8, SLC6A4, PTGS2, EGR3, PILRA

MDSC	CCR2, CD14, CD2, CD86, CXCR4, FCGR2A, FCGR2B, FCGR3A, FERMT3, GPSM3, IL18BP, IL4R, ITGAL, ITGAM, PARVG, PSAP, PTGER2, PTGES2, S100A8, S100A9
Monocyte	ASGR2, CFP, ASGR1, CD1D, UPK3A, ACTG1, ANXA5, ATP6V1B2, CFL1, DAZAP2, CTBS, EMR4P, HIVEP2, MARCKSL1, MBP, MMP15, PNPLA6, TMBIM6, PQBP1, TEX264, IKZF1
Natural killer cell	AKT3, AXL, BST2, CDH2, CRTAM, CSF2RA, CTSZ, CXCL1, CYTH1, DAXX, DGKH, DLL4, DPYD, ERBB3, F11R, FAM27A, FAM49A, FASLG, FCGR1A, FN1, FSTL1, FUCA1, GBP3, GLS2, GRB2, LST1, BCL2, CDC5L, FGF18, FUT5, FZR1, GAGE2, IGFBP5, KANK2, LDB3
Natural killer T cell	BTN2A2, CD101, CD109, CNPY3, CNPY4, CREB1, CRTC2, CRTC3, CSF2, KLRC1, FUT4, ICAM2, IL32, LAMP2, LILRB5, KLRG1, HSPA4, HSPB6, ISM2, ITIH2, KDM4C, KIR2DS4, KIRREL3, SDCBP, NFATC2IP, MICB, KIR2DL1, KIR2DL3, KIR3DL1, KIR3DL2, NCR1, FOSL1, TSLP, SLC7A7, SPP1, TREM2, UBASH3A, YBX2, CCDC88A, CLEC1A, THBD, PDPN, VCAM1, EMR1
Neutrophil	CREB5, CDA, CHST15, S100A12, APOBEC3A, CASP5, MMP25, HAL, C1orf183, FFAR2, MAK, CXCR1, STEAP4, MGAM, BTNL8, CXCR2, TNFRSF10C, VNN3
Plasmacytoid dendritic cell	CBX6, DAB2, DDX17, HIGD1A, IDH3A, IL3RA, MAGED1, NUCB2, OFD1, OGT, PDIA4, SERTAD2, SIRPA, TMED2, ENG, FCAR, IGF1, ITGA2B, GABARAP, GPX1, KRT23, PROK2, RALB, RETNLB, RNF141, SEC14L1, SEPX1, EMP3, CD300LF, ABTB1, KLHL21, PHRF1

Supplementary Table 5. Spearman correlation coefficients and p values between glycolysis-related key DEGs and immune cell infiltration levels

Immune cell	Gene	Spearman_rho	P_value
Activated B cell	CLDN3	-0.07228	0.097348111
Activated CD4 T cell	CLDN3	0.738877	4.69E-18
Activated CD8 T cell	CLDN3	-0.74424	1.18E-21

Activated dendritic cell	CLDN3	0.725469	2.09E-16
CD56bright natural killer cell	CLDN3	-0.45206	1.39E-10
CD56dim natural killer cell	CLDN3	-0.50348	6.59E-14
Central memory CD4 T cell	CLDN3	0.709049	4.29E-14
Central memory CD8 T cell	CLDN3	0.810547	2.37E-17
Effector memeory CD4 T cell	CLDN3	-0.31881	0.000620532
Effector memeory CD8 T cell	CLDN3	0.879199	3.96E-25
Eosinophil	CLDN3	-0.76302	3.01E-18
Gamma delta T cell	CLDN3	0.74745	1.15E-16
Immature B cell	CLDN3	0.379372	6.90E-06
Immature dendritic cell	CLDN3	0.728035	4.16E-16
Macrophage	CLDN3	-0.41851	6.83E-08
Mast cell	CLDN3	0.461488	3.01E-09
MDSC	CLDN3	0.89226	6.12E-23
Memory B cell	CLDN3	0.074996	0.07868384
Monocyte	CLDN3	0.313788	6.19E-05
Natural killer cell	CLDN3	-0.13615	6.88E-05
Natural killer T cell	CLDN3	0.193087	0.109629352
Neutrophil	CLDN3	-0.22428	6.67E-08
Plasmacytoid dendritic cell	CLDN3	-0.62382	6.64E-14
Regulatory T cell	CLDN3	0.818083	8.03E-18

T follicular helper cell	CLDN3	0.850051	3.47E-22
Type 1 T helper cell	CLDN3	0.778278	5.66E-13
Type 17 T helper cell	CLDN3	-0.4186	3.99E-10
Type 2 T helper cell	CLDN3	-0.11957	7.16E-05
Activated B cell	CD44	0.023172	0.135422082
Activated CD4 T cell	CD44	0.810843	3.55E-20
Activated CD8 T cell	CD44	-0.7738	7.21E-24
Activated dendritic cell	CD44	0.756761	2.49E-17
CD56bright natural killer cell	CD44	-0.4486	2.65E-11
CD56dim natural killer cell	CD44	-0.49735	2.46E-14
Central memory CD4 T cell	CD44	0.72865	1.03E-14
Central memory CD8 T cell	CD44	0.871175	3.98E-19
Effector memeory CD4 T cell	CD44	-0.23529	0.001229418
Effector memeory CD8 T cell	CD44	0.882181	2.57E-22
Eosinophil	CD44	-0.78372	3.75E-19
Gamma delta T cell	CD44	0.76994	1.35E-16
Immature B cell	CD44	0.459886	3.36E-06
Immature dendritic cell	CD44	0.77324	4.49E-17
Macrophage	CD44	-0.34372	3.68E-07
Mast cell	CD44	0.559069	1.68E-10
MDSC	CD44	0.930845	1.52E-26

Memory B cell	CD44	0.218108	0.034408415
Monocyte	CD44	0.396339	3.58E-05
Natural killer cell	CD44	-0.13548	3.50E-05
Natural killer T cell	CD44	0.197346	0.081343728
Neutrophil	CD44	-0.23514	2.59E-08
Plasmacytoid dendritic cell	CD44	-0.69236	1.32E-15
Regulatory T cell	CD44	0.888652	2.16E-20
T follicular helper cell	CD44	0.886408	2.99E-24
Type 1 T helper cell	CD44	0.801774	1.96E-12
Type 17 T helper cell	CD44	-0.45253	6.75E-11
Type 2 T helper cell	CD44	-0.02406	7.09E-05
